# The psychometric properties of Binge Eating Scale among overweight college students in Taiwan

**DOI:** 10.1186/s40337-023-00774-3

**Published:** 2023-03-25

**Authors:** Huey-Yeu Yan, Fu-Gong Lin, Mei-Chih Meg Tseng, Yue-Lin Fang, Hung-Ru Lin

**Affiliations:** 1 PhD Program, School of Nursing, National Taipei University of Nursing and Health Sciences, Taipei City, 112303 Taiwan; 2 Department of Nursing, University of Kang Ning, Taipei City, 114311 Taiwan; 3 Department of Optometry, Asia University, Taichung, 413305 Taiwan; 4 Department of Psychiatry, School of Medicine, College of Medicine, Taipei Medical University, Taipei City, 110301 Taiwan; 5 Department of Psychiatry, Shuang Ho Hospital, Taipei Medical University, New Taipei City, 235041 Taiwan; 6 Division of General Surgery, Department of Surgery, Shin Kong Wu Ho-Su Memorial Hospital, No.95, Wenchang Rd., Shilin Dist., Taipei City, 111045 Taiwan; 7 School of Nursing, National Taipei University of Nursing and Health Sciences, No. 365, Mingde Rd., Beitou Dist., Taipei City, 112303 Taiwan; 8 Department of Psychiatry, National Taiwan University College of Medicine, Taipei City, 100233 Taiwan

**Keywords:** Binge eating, Overweight, College students, Psychometric properties, Cross cultural

## Abstract

**Background:**

The Binge Eating Scale (BES) is a widely used measuring tool to assess binge eating problems in Western countries. However, the psychometric properties of such scales among cross-cultural youth groups are insufficient, and the factor structure continues to be debated; therefore, further research is needed. The aim of this study was to examine the properties of BES among overweight college students in Taiwan.

**Methods:**

A cross-sectional design and convenience sampling were adopted to recruit 300 overweight students from five universities. A translated Traditional Chinese version of BES was used for the survey, and the validity of the scale was tested using the Confirmatory Factor Analysis (CFA) and Bulimic Investigatory Test, Edinburgh (BITE). The reliability was evaluated using internal consistency and test–retest reliability.

**Results:**

The CFA results showed a reasonable model fit. The first-order two-factor model was consistent with that of the original BES and significantly correlated with the criterion of BITE score. Cronbach’s α value, representing internal consistency reliability, and the intraclass correlation coefficient of repeated measures made one month apart were both 0.83, indicating good reliability and stability. Significant correlations were observed between the BES score and sex and BMI; however, no correlation was observed between BES scores and age.

**Conclusion:**

The BES presents sound psychometric properties, has good cross-cultural applicability, and can be used as a first-line screening tool by mental health professionals to identify the severity of binge eating behavior among overweight college students in Taiwan. It is recommended that participant diversity and obesity indicators be incorporated into the scale in the future to establish a universal psychometric tool.

**Supplementary Information:**

The online version contains supplementary material available at 10.1186/s40337-023-00774-3.

## Abstract (summary)

The Binge Eating Scale (BES) is a screening tool that has been widely used to assess binge eating problems in Western countries. The current study aimed to test the validity and reliability properties of the BES among overweight college students in Taiwan. This research involved 300 overweight and obese college students while using a traditional Chinese-translated questionnaire in the survey and analyzed with subjective and scientific statistics methods afterward. The results indicated that BES has good cross-cultural applicability and can be used as a first-line measuring tool by mental health professionals to identify the severity of binge eating behavior among overweight or obese college students in Taiwan.

**Supplementary Information:**

The online version contains supplementary material available at 10.1186/s40337-023-00774-3.

## Background

Binge eating disorder (BED) is the most common type of eating disorder and is one mental illness with rather high morbidity [1]. A national research report in the U.S. indicated that the lifetime prevalence of BED was 0.85% among adults over the age of 18 years in the United States (n = 36,306). This prevalence was higher than that of two other eating disorders: anorexia nervosa (AN), at 0.8%, and bulimia nervosa (BN), at 0.28% [2]. The primary symptom of BED according to DSM-5 criteria is recurrent episodes of binge eating, characterized by eating a large amount of food in a short period (usually two hours) and a sense of losing control over eating [1]. Due to the highest incidence rate found at the age of 14 and 19–24 years old and mostly related to body weight and emotional distress, it seriously impacts physical and mental health [3–6].

Influenced by Western eating habits and culture in the past two decades, binge eating problems have emerged in Taiwan. A clinical study found that as high as 15.9% (*n* = 189) of those who participated in weight-loss courses in hospitals revealed binge eating symptoms, most of whom were young and had early-onset obesity and psychological distress [7]. Another study also found that 42% of people with obesity whoreceived clinical weight loss treatment (*n* = 841) were diagnosed with at least one mental disorder, of which eating disorders, mood disorders, and anxiety disorders accounted for the highest proportions [8]. In addition, another study analyzed National Health Insurance data from 2002 to 2013 and found that the incidence rate of BN, which has the same binge-eating symptoms as BED, also increased year by year, with 6.1 cases per 100,000 samples and an average increase of 4.96% each year until 2009, dropping slightly to 4.95% afterward. The highest incidence rate (51.3%) was found in the age group of 20–29 years old (5406 cases) [9]. Compared with the peak onset cited above (14 and 19–24 years old), the age group with the highest incidence rate in Taiwan appeared to be more than ten years older than the United States. It was estimated that a group of young people had suffered from binge eating but were not used to accessing medical help [9]. In addition, this hidden group was mostly at college age and underwent great academic pressure, which may trigger eating disorder relapse [10]. Therefore, it is imperative to develop an appropriate traditional Chinese version of the BES screening tool in Taiwan for early detection of BED and other eating disorders and assistance with psychological treatment or in conjunction with other medical treatments.

The Binge Eating Scale (BES) is a tool used to assess binge eating problems among obese persons. It consists of 16 items of self-report questions. This scale was developed by Gormally et al. [11] based on years of clinical experience and psychometric data in treating patients with binge eating. The content of the scale included 8 items describing behavior manifestations and 8 items about feelings and cognitions surrounding a binge episode. Each item contains three to four levels of symptom descriptions with 0–2 or 0–3 points. The total score ranged from 0 to 46. The higher the score, the more severe the binge eating problems. To distinguish levels of binge-eating severity, Marcus et al. [12] set three cutoff scores by subtracting or adding half of the standard deviation to the mean BES score and created three score ranges of ≤ 17 = none to mild, 18–26 = moderate, or ≥ 27 points = severe binge eating problems. This scale, with an internal consistency reliability α value ranging from 0.80 to 0.93, was widely used, not only by people with overweight and obesity but also by community residents and college students from different countries, for example, being translated and tested in France (by French), Portugal (by Portuguese), Spain (by Spanish), Malaysia (by Malay), Lebanon (by Arabic), and China (by Simplified Chinese) [5, 13–17].

Although the BES has been widely used in Western countries and is convenient to score and easy to administer, the composition dimension is controversial when applied cross-culturally. For example, studies in China, Puerto Rico, France, and Spain showed that the best goodness of fit for the translated questionnaire was a one-factor model [5, 13, 14, 16], which is different from the original version. In addition, when assessing reliability, some studies used the Pearson correlation coefficients to analyze repeated measures of the same sample [14, 16], which may not clearly reflect the correlation and consistency between the two levels of measurement [18]. In addition, the interval between tests was not clearly stated, which may affect the test–retest reliability results. Although one study by China translated the original BES into a Simplified Chinese version of BES (SC-BES), Traditional Chinese is still the official language in Taiwan [19], so people may have problems reading and understanding the SC-BES correctly. In addition, the glyph images of certain simplified words are prone to mistakes, and certain simplified words are unable to present unique connotations and significance [20]. They may not fit the cultural and habitual needs in Taiwan [21]. Furthermore, the age of participants in the SC-BES ranged from 12 to 18, so we evaluated a different group from this study. In light of the prevalence of overweight and obesity in the 18–24-year-old college stage in Taiwan being considerably high (29.3%) [22] but lacking applicable assessment tools, this study aimed to test the psychometric properties of a Traditional Chinese version of BES (TC-BES; see Additional file 1) by establishing its factor structure, internal consistency, and construct validity in overweight college students. It is hoped to develop and construct a reliable and valid BE screening tool for mental health professionals’ reference and usage.

## Methods

### Participants

This study adopted a cross-sectional design and convenience sampling method to recruit 300 students with overweight or obesity from five universities in Taiwan based on Asian anthropometric standards and cultural background. The inclusion criteria were as follows: (1) young people aged 18–24; (2) who had a body mass index (BMI) ≥ 24; and (3) who were willing to participate and sign a consent form (if younger than 20 years old, a parent's signature on a parental consent form was required). The exclusion conditions included (1) pregnancy; (2) breastfeeding; (3) a history of severe mental illness diagnosed by a physician; and (4) refusal to participate in this study.

The sample size was calculated based on the recommendation by Anderson and Grebing [23] that each variable in the factor analysis required at least 10–20 samples. Thus, this study invited 335 potential participants, among whom 310 were willing to participate. After excluding 10 people who did not meet the inclusion criteria (BMI < 24), a total of 300 subjects, 215 women (71.7%) and 85 men (28.3%), participated in the study. The participants' ages ranged from 18 to 24 years, with a mean age of 20.37 (SD = 1.31). Their BMI ranged from 24.01 to 49.67 kg/m^2^ with a mean of 29.82 (SD = 4.63). Among the participants, 27.0% were categorized as overweight (≥ 24 to  < 27), 29.0% as mildly obese (≥ 27 to  < 30), 28.7% as moderately obese (≥ 30 to  < 35), and 15.3% as severely obese (≥ 35).

### Measures

#### Binge Eating Scale (BES)

This study used BES as the research instrument. With the consent of the original author Jim Gormally, and based on the recommendations by Streiner et al. [24], the BES was translated into Mandarin Chinese using the following steps: (1) Forward translation: Two bilingual Taiwanese (one had a psychology background) translated BES into Chinese separately; (2) Comparison of the translations and synthesis version: The research team discussed the first drafts with translators and integrated them into a preliminary version (PV); (3) Backward translation/Blind back-translation: Two professional translators translated the PV back to the English version without reviewing the original questionnaire and then discussed with the research team and merged the drafts into one. The original author, Jim Gormally, was invited to assist in the review and revision. (4) Expert verification: Seven experts reviewed the semantic and conceptual equivalence between the Chinese PV and the original English scale using a four-point scale (1 point = very inappropriate; 4 points = very appropriate). The scale content validity index (scale-level CVI; S-CVI) was found to be 0.99, implying that the overall expert consistency was excellent. (5) Pilot test: Thirty overweight students were recruited to take a precursor test, and the internal consistency was found to be 0.88 (Cronbach’s α).

#### Bulimic Investigatory Test, Edinburgh (BITE)

The Bulimic Investigatory Test, Edinburgh (BITE) is a self-administered questionnaire designed to identify subjects with symptoms of bulimia or binge eating [25]. It consists of two subscales: (1) Symptom Scale (30 questions): To assess the current level of bulimia symptoms based on yes and no questions. One point was assigned to "yes", while 0 points were assigned to "no"; (2) Severity Scale (3 questions): Scores were assigned to questions 6, 7, and 27 according to the frequency of binge eating and compensatory behaviors. The total score was the sum of the numbers corresponding to the circled responses with a maximum of 39 points. Thus, the total possible score of BITE was 69 points. The validity criterion was set at 25 points. A score higher than 25 points was indicative of a severe current eating disorder and existing binge eating problems [13, 25].

The Chinese version of the BITE was translated by Tseng et al. [26] after obtaining the original author's consent. The internal consistency reliability as represented by Cronbach α values for the subscales were 0.95 and 0.77, while the ICC values were 0.87 and 0.88. Based on the recommendation from previous studies [9, 26, 27], BITE scores 26–28 were used as the validity criterion. With the above settings, the sensitivity, specificity, positive predictive value, negative predictive value, and diagnostic efficiency were all 1. On the other hand, the diagnostic efficiency dropped to 0.98 or 0.99 when set to 21–25 or 29, respectively [26]. To avoid false negative errors and achieve the study goal of early detection, the cutoff point was set to 26. The reliability and diagnosability of this scale were good. The scale is currently considered an appropriate self-administered questionnaire that could assist in the diagnosis of bulimia in practice in Taiwan [7, 26, 27] and is suitable for use as a relative criterion with the assessment tool in this research.

#### BMI

The participants' BMIs were determined using a body fat meter (model, OMRON HBF-216, medical instrument certificate No. 000704) with the logged individual's height and the actual measured weight, calculated automatically by the instrument in real-time.

### Procedure

After receiving IRB approval (TCHIRB-10910001-E), data collection was conducted at five different universities. Most participants, 260 out of 300 (86.7%), were from four universities in the metropolitan area of northern Taiwan. The remaining 40 participants (13.3%) came from a university in the suburbs of central Taiwan. During the data collection process, the investigator first explained study objectives and procedures to potential participants both in writing and verbally. Data collection was conducted in a face-to-face manner and only initiated after obtaining the participants’ and their guardians’ written consent. Participation was voluntary. The questionnaires were self-administered, and the data were kept anonymous. Anyone who refused to take part was excluded before data collection. The participants completed questionnaires in a quiet campus environment, such as meeting rooms, libraries, and classrooms. The investigator checked the completeness of the questionnaire on-site when it was returned. The participants were asked to complete unanswered questions instantly. We set the test–retest reliability to 0.8 based on a previous study [14]. The study adopted 1.5 times the required sample size (19) to offset any potential attrition rate. As a result, this study used a subsample size of 30 to ensure test–retest reliability, and a sample suit for the study criteria was adopted to invite 30 participants to repeat the survey after one month. The data collection period was from February 11, 2011, to May 31, 2021.

### Statistical analysis

SPSS 22 and LISREL 8.51 statistical software were used in data analysis. A CFA was performed in construct validity testing based on the data of Bagozzi and Yi [28] and of Bollen [29] with the following model fit indices used: (1) Chi-square (χ^2^); (2) χ2 with degree of freedom (χ2/df; ideal value = 1 ~ 3); (3) goodness-of-fit index (GFI; ideal value > 0.90); (4) root mean square error of approximation (RMSEA; good fit < 0.05; fair fit = 0.05–0.08); (5) standardized root mean square residual (SRMR; good fit < 0.05; fair fit = 0.05–0.08); (6) adjusted goodness-of-fit index (AGFI; ideal value > 0.90; acceptable value > 0.8); (7) comparative fit index (CFI; ideal value > 0.95); and (8) nonnormed fit index (NNFI; ideal value > 0.95). Spearman's rank correlation coefficient (rho) was performed to test concurrent validity with BITE (> 0.5–0.75 indicating a moderate to a good relationship) [30], and the receiver operating characteristic curve (ROC) was used to describe the instrument’s performance. Reliability was determined by the internal consistency of Cronbach's alpha, and the test–retest reliability was assessed using the intraclass correlation coefficient (ICC). In addition, demographic data were analyzed using descriptive and correlational statistics.

## Results

### Participants descriptive

The mean BES score of the 300 participants was 10.67 (SD = 6.66; range: 0–34). According to the cutoffs recommended by Marcus et al. [12], 248 (82.7%) participants who scored less than or equal to 17 points were considered to have no to mild binge eating issues. Forty-six participants (15.3%) had moderate binge eating with scores ranging from 18 to 26, while 6 participants (2%) had severe binge eating with scores ≥ 27. In addition, the mean BITE score was 10.20 (SD = 6.72; range: 1–40). The cutoff for the Chinese version of the BITE was 26 points. Among the participants, 291 (97%) scored less than 26 points, indicating an unlikely presence of an eating disorder or existing binge eating. Alternatively, nine participants (3%) had scores ≥ 26 points, indicating the likely presence of bulimia nervosa.

### Factor structure

CFA was used to evaluate whether the latent variables of the TC-BES components were consistent with the empirical data. The maximum likelihood (ML) estimation method was adopted for model estimation. Provided there were no offending estimates, the overall model fit test result was χ^2^ = 222.23 (*p* < 0.001), df = 103, χ^2^/df = 2.16 (< 3 is considered good fit), GFI = 0.91 (> 0.90 is considered good fit), AGFI = 0.89 (> 0.8 and not exceeding the GFI value is considered acceptable), CFI = 0.95 and NNFI = 0.94 (> 0.9 is a good fit), SRMR = 0.06 and RMSEA = 0.06 (values between 0.05 and 0.08 are fair fit). Therefore, it was confirmed that the first-order two-factor model had fair construct validity, and the factor structure and correlational relationship between variables were reasonable and acceptable.

### Concurrent validity

**Fig. 1:**
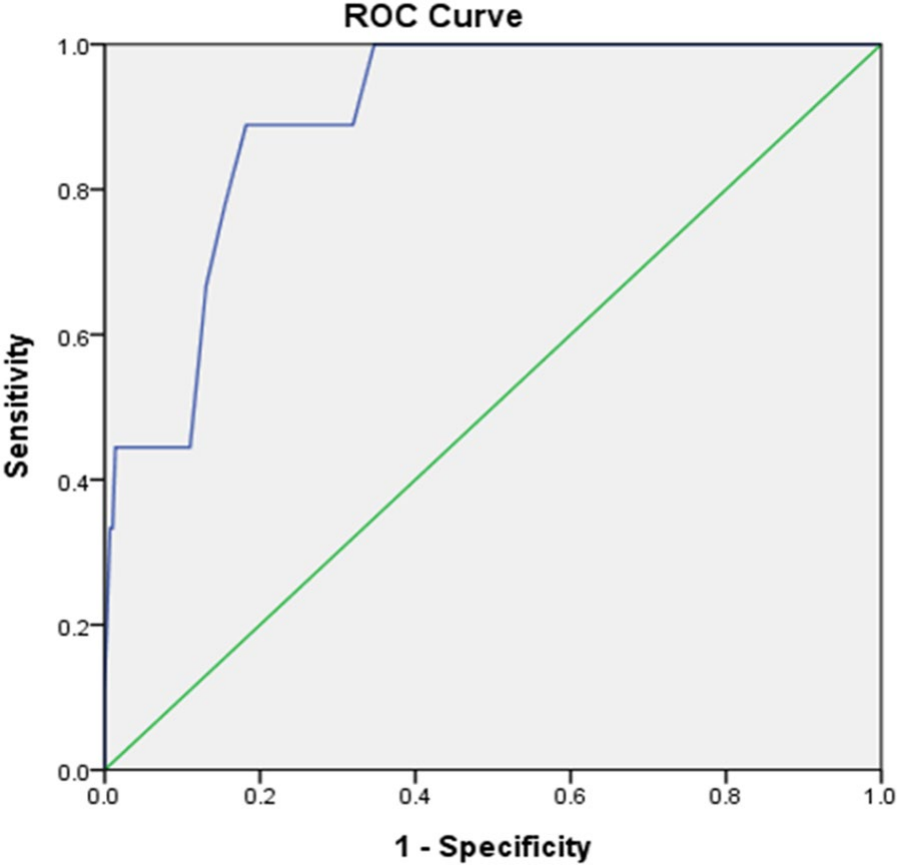
ROC curve showing the accuracy of BES as a screening tool for binge eating behavior (AUC = 0.9; 95% CI = 0.83–0.97)

Residual analysis suggested that *p* < 0.001, and the data were not normally distributed. Therefore, Spearman's rank correlation coefficient was adopted to determine the correlation between BES and BITE, which revealed a significant positive correlation (r_s_ = 0.69, *p* < 0.01). Next, the C-BITE cutoff score of 26 was used for diagnostic evaluation. The results of the receiver operating characteristic curve (ROC) analysis (Fig. 1) showed that the area under the curve (AUC) = 0.9 (95% CI = 0.83–0.97), which was higher than the standard of 0.7. In addition, the significance test revealed that *p* < 0.001, which suggested that the BES scale exhibited a significant effect in predicting symptoms. Subsequently, the Youden index was utilized to determine the best TC-BES cutoff score, which showed a sensitivity of 0.889, a specificity of 0.818, and an optimal cutoff score of 17 points.

### Internal consistency reliability

The result of the TC-BES internal consistency reliability test, as indicated by Cronbach's α, was 0.83. After removing the individual item, Cronbach's α ranged from 0.80 to 0.83, indicating good correlations between items in the scale. In addition, the homogeneity of the questions was tested using the corrected item-total correlation (r _tot_). The correlation coefficients of the 16 items were all significantly higher than the standard value of 0.3 (*p* < 0.01), except for questions 6 and 13 (r = 0.26). Table 1 presents means, standard deviations, and percentages of BE symptoms (i.e., scores of 1, 2, or 3), corrected item-total correlations (r _tot_), and Cronbach's α values if any BES items were deleted.

**Table 1:** Mean scores (M), standard deviations (SD), percentages of symptoms (%), corrected item-total correlation (r_tot_), and Cronbach's alpha (α) if any BES items were deleted

Items	M	SD	%	r _tot_	α if item deleted
Item 1	0.50	0.80	37.70	0.33	0.82
Item 2	1.28	0.94	75.70	0.36	0.82
Item 3	0.47	0.82	32.70	0.57	0.81
Item 4	0.14	0.51	7.00	0.31	0.82
Item 5	0.68	0.61	61.00	0.47	0.82
Item 6	0.81	0.75	67.70	0.26	0.83
Item 7	0.97	1.27	38.30	0.51	0.81
Item 8	1.04	0.85	68.30	0.51	0.81
Item 9	0.91	0.76	67.70	0.49	0.81
Item 10	0.52	0.79	34.70	0.64	0.80
Item 11	0.61	0.56	57.70	0.52	0.81
Item 12	0.48	0.73	36.00	0.39	0.82
Item 13	0.22	0.69	9.30	0.26	0.83
Item 14	0.94	0.88	63.70	0.41	0.82
Item 15	0.67	0.78	53.00	0.47	0.81
Item 16	0.44	0.63	36.70	0.58	0.81

Sample Size (n = 300), % = Percentage of the samples selecting 1, 2, or 3

**Table 2:** Results of ICC calculation in SPSS using 2-way mixed-effects model, single measurement, absolute-agreement

	Intraclass correlation	95% Confidence interval		F test with true value 0			
		Lower bound	Upper bound	Value	df1	df2	Sig
Single measures	0.830	0.652	0.918	12.294	29	29	0.000

### Test–retest reliability

Thirty participants were randomly selected from the total sample (n = 300) to answer a second administration of the BES to test the scale’s temporal stability. The coefficient of stability was estimated using a two-way mixed-effects model based on a single measurement type and the absolute agreement relationship. The ICC for the results of the scale repeated one month after the first test was 0.83 (*p* < 0.01), which was greater than the standard of 0.75 [18], indicating good stability and good reliability (Table 2).

### Correlation with sex, age and BMI

Spearman’s rank correlation coefficient was adopted to determine the correlation between the BES score and various demographic variables. The correlation coefficients (r_s_) between the BES score and sex and BMI were − 0.14 and 0.12, respectively, and both reached the significance level (*p* < 0.05). In contrast, age (r_s_ = − 0.02) had no significant relationship with the BES score.

## Discussion

In this study, LISREL was used to test the construct validity of the C-BES. The results from CFA revealed that the overall model fit was good, and there was a good and acceptable relationship between the factor structures. Although the χ^2^ value showed a significant difference (*p* < 0.01), it is important to note that the chi-square test is sensitive to sample size, and most differences will appear statistically significant when the sample size is large [31–33]. A further examination at χ^2^/df showed that the adjusted value was 2.66, which was smaller than the standard value of 3 [34]. In this study, most of the indices met the criteria of good model fit. The testing results were in line with the original version of the scale and consistent with findings by researchers from other countries [15, 17]. When compared with other studies, our study provided two-factor results instead of one-factor results [5, 16], and different results could also come from recruiting participants of different age groups. We recommend testing participants of various ages and BMI in future studies to develop a BE screening tool that applies to the general population.

In terms of content validity testing, the original author of the scale, Dr. Gormally, was invited to assist in reviewing the content of the backward translation. Seven Taiwanese experts were also invited to help with the review of the questionnaire translation, including two psychiatrists with expertise in binge eating disorders, three mental health experts specializing in eating disorder patient care, and two linguistics professors. After two consensus meetings and revisions, the content validity indices (item-level content validity index; I-CVI) for all questions (16 items, a total of 62 options) were 1. Only three options had indices of 0.86, which were well above the standard value of 0.78 for I-CVI [35]. This result showed that the translated scale was very representative, and the expert content validity was excellent. This research advocates the need to have five to seven expert reviewers in any future translation of study instruments. Furthermore, the expertise of the reviewers should be aligned with the area of the review to achieve effectiveness in cross-cultural language communication [20, 36].

In testing criterion-related validity, clinical evidence showed that BED was closely related to bulimia nervosa (BN), and both have obvious binge eating symptoms. Patients with BN also regularly exhibit compensatory behaviors, such as rigorous exercise, induced vomiting, laxative use, or fasting [1, 37, 38]. This study used BITE, the scale for diagnosing bulimia, as the key indicator for assessing criterion-related validity. The testing results were consistent with the empirical data, indicating a positive and significant relationship. The results support the C-BES as a validity tool for assessing BE behaviors among overweight or obese college students in Taiwan.

In terms of reliability, the internal consistency of the C-BES was good (α = 0.83), well above the acceptable value of 0.7, indicating good reliability [39]. Although the corrected item-total correlations (r_tot_) for items 6 and 13 were 0.26, the two items were retained in the model, as they were essential in identifying important characteristics of BED, including the extent of eaters' guilt after overeating and dietary abstinence between meals. The other reason was that the removal of individual questions did not increase the internal consistency of the overall scale. It is recommended that item clarity be further enhanced in terms of semantics to increase the level of discernment in the future. The results of the test–retest reliability were quite good. The scores of the two repeated measurements, with a one-month interval, had a significant correlation, and the ICC exceeded the reference value of 0.8 [18]. The C-BES had good reliability and stability and can be employed in large-scale surveys cost-effectively.

Demographic data suggested that subjects’ BES scores were significantly associated with their gender and BMI, which is consistent with the findings of most Western studies [40–42]. In addition, a recent national survey on young adults in the United States (n = 14,322; aged between 18 and 24 years) found that the prevalence of binge eating among overweight or obese individuals was substantially higher than that among normal or underweight individuals. More specifically, the prevalence was 29.3% versus 15.8% among women and 15.4% versus 7.5% among men. Subsequent logistic regression analysis indicated that the risk (odds ratio) of women developing binge eating was 2.32 times that of men (95% CI = 2.05–2.61) [43]. This result revealed the need for school health units to focus on the binge eating problem among college students and prioritize overweight or obese women for screening.

Since this study focused on college students, the inclusion criteria limited the age of participants to 18–24 years old. Such a narrow range in age may cause an insignificant difference in statistical testing. The results of this study support the above viewpoints and recommend that future studies be conducted to thoroughly explore the psychological factors associated with binge eating among people of diverse cultures and different genders to help develop positive coping strategies for regulating emotional stress.

### Limitations

This study adopted convenience sampling to survey young students from five colleges and universities characterized by their focus on developing healthcare programs. As most of these schools are in the metropolitan area of northern Taiwan, this study may not reach all young people with binge eating disorders. It is suggested that future studies should include diversified participants, schools of higher heterogeneity, or even those of different age groups to enhance the applicability and popularity of the scale. In addition, this study defined overweight and obese individuals based on World Health Organization [WHO] recommendations. The diagnostic criteria for overweight (BMI ≥ 24 kg/m^2^) and obese (BMI ≥ BMI 27 kg/m^2^) were based on data published by Taiwan’s Ministry of Health and Welfare [22]. The cutoff BMI values were different from the standards of Western countries. Due to racial differences and other discrepancies in disease-related conditions, how to clearly define the criteria for overweight and obesity is a highly discussed topic. It is recommended that future studies adopt multiple indicators, such as the body fat ratio, waist circumference, or waist-hip ratio (WHR), to diagnose overweight and obesity holistically.

## Conclusion

The aim of this study was to examine the psychometric properties of the BES scale among overweight college students in Taiwan. The original BES was translated into Mandarin Chinese by our research team and was tested with good reliability and stability. Diagnostic evaluation using BITE with 26 as the cutoff score created an optimal C-BES cutoff score of 17 and a high level of both sensitivity (88.9%) and specificity (81.8%), indicating that the scale could effectively predict BE symptoms. The results indicate that the BES presents sound psychometric properties, has good cross-cultural applicability, and can be used as a first-line screening tool by mental health professionals to identify the severity of binge eating behaviors among overweight college students in Taiwan. It is recommended that participant diversity and obesity indicators be incorporated into the scale in the future to establish a universal psychometric tool.

## Supplementary Information

**Additional file 1**. Appetite Scale.

## Data Availability

All data generated and/or analyzed during the current study are available from the corresponding author on reasonable request.

## Abbreviations

AGFI: Adjusted Goodness-of-Fit Index
AUC: Area Under the Curve
BED: Binge Eating Disorder
BES: Binge Eating Scale
BITE: Bulimic Investigatory Test, Edinburgh
BMI: Body Mass Index
BN: Bulimia Nervosa
C-BES: Chinese Version of BES
CFA: Confirmatory Factor Analysis
CFI: Comparative Fit Index
DSM-5: Diagnostic and Statistical Manual of Mental Disorders, Fifth Edition
GFI: Goodness-of-Fit Index
ICC: Intraclass Correlation Coefficient
I-CVI: Item-Level Content Validity Index
ML: Maximum Likelihood
NNFI: Non-Normed Fit Index
RMSEA: Root Mean Square Error of Approximation
SRMR: Standardized Root Mean Square Residual
WHR: Waist-Hip Ratio
