# Supplementary material for: The psychometric properties of Binge Eating Scale among overweight college students in Taiwan
Source: J Eat Disord. 2023 Mar 25;11:47. doi: 10.1186/s40337-023-00774-3 (PMC10039566; doi:10.1186/s40337-023-00774-3)
Supplement: Supplementary file 1 — Additional file 1. Appetite Scale. [file 40337_2023_774_MOESM1_ESM.docx]

**第一部分:飲食習慣調查表**

- 請您詳細閱讀以下題目的句子，選出最符合您個人行為/感受的陳述，並在該選項數字前打勾☑，這些問題都是關乎個人飲食習慣的陳述，請您放心填答!

第1題

- - 1.和別人相處時，我不會特別注意自己的體重或體型。
  - 2.我擔心別人怎麼看我的外表，但這並不會影響我的自信。
  - 3.我在意自己的外表和體重，且這讓我對自己感到失望。
  - 4.我非常在意自己的體重，覺得很羞愧和討厭自己，所以會盡量避開社交活動。

第2題

- - 1.對我而言，放慢吃東西的速度，毫不困難。
  - 2.雖然我好像很快的將食物嚥下，卻不會因為吃多而感到肚子脹。
  - 3.有時我容易吃得很快，之後會感到太飽不適。
  - 4.我習慣大口快速吞下食物，沒有仔細咀嚼，之後會因為吃太多而撐得難受。

第3題

- - 1.我可以掌控自己吃東西的慾望。
  - 2.我比一般人無法掌控自己的進食。
  - 3.我對於掌控自己吃東西的慾望，感到無能為力。
  - 4.我實在無力掌控自己的進食，對於試著控制它，已經變得非常絕望。

第4題

- - 1.無聊的時候，我沒有吃東西的習慣。
  - 2.無聊的時候，有時我會吃東西，但常可藉由「忙碌」，讓自己不再想著食物。
  - 3.無聊的時候，我經常會吃東西，但偶爾也會安排活動，讓自己不再想著食物。
  - 4.無聊的時候，我有吃東西的習慣，很難改變這種慣性。

第5題

- - 1.我吃東西時，通常是肚子餓了。
  - 2.偶爾我會因為衝動而吃東西，即使自己不餓。
  - 3.為了滿足飢餓感，我有定時吃東西的習慣，即使不是自己喜歡的食物，甚至當時不餓。
  - 4.即使肚子不餓，我也會想吃東西，為了滿足口慾，有時我會先吃再吐出來，以免體重增加。

第6題

- - 1.我吃得太多之後，並沒有任何罪惡感或自我厭惡。
  - 2.我吃得太多之後，偶爾會有罪惡感或自我厭惡。
  - 3.我吃得太多之後，通常都會感到強烈的罪惡感或自我厭惡

第7題

- - 1.我在節食時候，即使吃得太多後，仍然可以控制自己的飲食。
  - 2.我在節食時候，有時吃了一項「禁忌食物」，覺得自己「搞砸了計畫」，就會吃得更多。
  - 3.我在節食時候，當吃得過量時，經常會對自己說:「反正已經搞砸了計畫，乾脆就繼續吃吧!」，因此我會吃得更多。
  - 4.我習慣在進行一項嚴格的節食計畫時，就會開始狂吃，以至於我的生活不是大吃大喝， 就是飢餓度日。

第8題

- - 1.我很少吃過多食物，讓自己太飽不舒服。
  - 2.大約一個月一次，我會吃得過量，感覺肚子很撐。
  - 3.一個月總有幾次，在正餐或點心時刻，我會吃下大量的食物。
  - 4.我經常吃得太多，吃完後會感到很不舒服，甚至有時想吐。

第9題

- - 1.在一般情況下，我從食物中攝取的熱量是平穩的，不會忽高忽低。
  - 2.有時在我吃太多食物之後，為了平衡攝取過多的熱量，自己會嘗試少吃，甚至不吃。
  - 3.我習慣在晚上飲食過量，這種慣性讓自己早上不餓，但夜晚吃得太多。
  - 4.我成年以後，在吃太多食物之後，就會有長達一周的挨餓期，似乎我的生活不是大吃大喝，就是飢餓度日。

第10題

- - 1.我通常能隨時停止不吃，知道何時該「適可而止」。
  - 2.偶爾我會經歷到無法控制的強迫性進食。
  - 3.我常會經歷到無法控制的強烈進食慾望，但有時候能控制住它。
  - 4.我無法控制自己吃東西的慾望，害怕不能自主的停止進食。

第11題

- - 1.我吃飽以後，能停止進食。
  - 2.我吃飽以後，通常就能停止進食，偶爾吃過量會撐得難受。
  - 3.我一旦開始進食，就很難停下來，經常吃完一頓飯後就會撐得難受。
  - 4.我一旦開始進食，就無法停下來，有時候要用催吐、瀉藥或利尿劑來緩解吃撐的感覺。

第12題

- - 1.不論是獨自進餐或與他人共食 (家庭或社交聚餐)，我吃的量都差不多。
  - 2.有時我和別人一同用餐，不會隨心所欲的吃，因為在意自己的進食。
  - 3.我經常在別人面前吃得很少，因為對於自己的進食，覺得很尷尬。
  - 4.我對自己吃得過多感到很羞愧，所以會挑選沒人看到的時候大吃，像是一位「躲在廚櫃裡的偷吃者」。

第13題

- - 1.我每日正常吃三餐，偶爾在兩餐之間吃零食。
  - 2.我每日正常吃三餐，但通常會在兩餐之間吃零食。
  - 3.當我吃太多零食時，就習慣略過正餐不吃
  - 4.我經常會一直吃個不停，沒有計畫性的飲食。

第14題

- - 1.我很少想過該如何控制不該有的進食慾望。
  - 2.至少有些時候，我的思緒會被如何控制進食慾望的想法所佔據。
  - 3.我經常花許多時間在想自己吃了多少或如何不要再吃了。
  - 4.似乎我大部分清醒時間都在想著吃或不吃，感覺自己一直在掙扎著不要吃。
  - 第15題
  - 1.我不常想著食物。
  - 2.我強烈渴望著食物，但只有持一會兒。
  - 3.有那麼幾天，我的思緒都在食物上，無法想別的事物。
  - 4.我經常整天都在想著食物，覺得自己是為了吃而活。

第16題

- - 1.我通常知道自己是否肚子餓了，並且會吃適量的食物來滿足自己。
  - 2有時不確定自己是否肚子餓了，在這種情況下，我不知道要吃多少食物來滿足自己。
  - 3.即使知道自己應該攝取多少卡路里，我仍然不知道該吃多少食物才算「正常」。
